# The potential role of DNA methylation as preventive treatment target of epileptogenesis

**DOI:** 10.3389/fncel.2022.931356

**Published:** 2022-07-22

**Authors:** Toni Christoph Berger, Erik Taubøll, Kjell Heuser

**Affiliations:** ^1^Department of Neurology, Oslo University Hospital, Oslo, Norway; ^2^Faculty of Medicine, University of Oslo, Oslo, Norway

**Keywords:** epilepsy, epileptogenesis, DNA methylation, gene expression, therapy, epigenetics, glia, inflammation

## Abstract

Pharmacological therapy of epilepsy has so far been limited to symptomatic treatment aimed at neuronal targets, with the result of an unchanged high proportion of patients lacking seizure control. The dissection of the intricate pathological mechanisms that transform normal brain matter to a focus for epileptic seizures—the process of epileptogenesis—could yield targets for novel treatment strategies preventing the development or progression of epilepsy. While many pathological features of epileptogenesis have been identified, obvious shortcomings in drug development are now believed to be based on the lack of knowledge of molecular upstream mechanisms, such as DNA methylation (DNAm), and as well as a failure to recognize glial cell involvement in epileptogenesis. This article highlights the potential role of DNAm and related gene expression (GE) as a treatment target in epileptogenesis.

## Introduction

**Figure 1:**
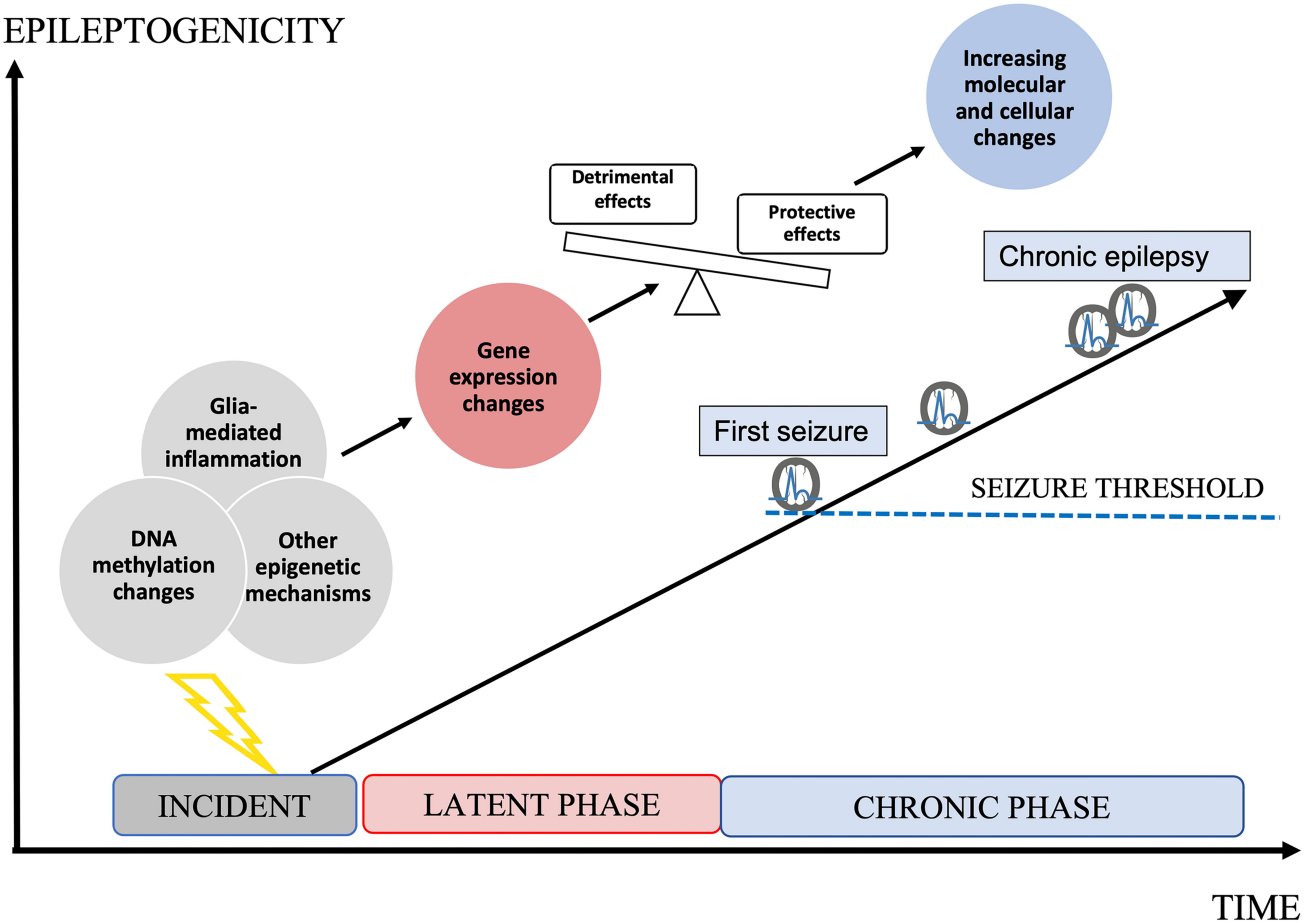
Epileptogenesis. Epileptogenesis describes the development of a brain becoming prone to generate epileptic seizures and further progression. An initial incident occurs to the brain, initiating the latent phase in which molecular and cellular changes increasingly develop. Epigenetic mechanisms, such as DNA methylation (DNAm) changes may determine changes in gene expression (GE) and by this cellular responses in both neurons and glial cells. Both protective and detrimental changes occur. Imbalance in favor to detrimental effects leads to increasing epileptogenicity up to the threshold of clinical seizures. During the chronic phase, epileptogenicity increases leading to the progression of clinical epilepsy, accompanied by increasing molecular and cellular changes. Both the latent phase and the chronic phase could be relevant to the development of anti-epileptogenic medication.

Epileptogenesis is the transformation of a physiologically functioning brain into an epileptic one, and the progression of manifested epilepsy (Pitkanen and Engel, 2014) (Figure 1). Although often initially not detectable clinically, this process is assumed to have a temporal and spatial starting point in the brain, involving an initial incident, such as trauma, hypoxia, infection, or complex febrile seizures. According to the current practical definition, this is followed by a latent phase devoid of clinical seizures, evolving to the chronic state that features the occurrence of spontaneous, and often progressive, epileptic seizures (Boison et al., 2013; Pitkanen and Engel, 2014). A prerequisite for the development of true anti-epileptogenic drugs preventing the emergence of clinical seizures, or stopping the worsening of chronic epilepsy, is to improve our understanding of the underlying pathological processes of epileptogenesis (Aronica and Gorter, 2007; Pitkanen and Lukasiuk, 2011; Loscher et al., 2013; Pitkänen et al., 2013; Löscher, 2020). Meanwhile, several pathological hallmarks of epileptogenesis are known. These are, for example, neuronal death, reactive gliosis, blood-brain barrier (BBB) disruption, axonal damage and sprouting, network reorganization, alteration of the extracellular matrix (ECM), and astrocyte uncoupling, as well as substantial changes of the molecular architecture of both neurons and glial cells (Tauck and Nadler, 1985; de Lanerolle et al., 1989, 2012; Houser, 1990; Houser et al., 1990; Mathern et al., 1995; Eid et al., 2005, 2019; Vezzani and Granata, 2005; Dityatev, 2010; Blumcke et al., 2013; Bedner et al., 2015; Patel et al., 2019; Bruxel et al., 2021). What is more delicate is the lack of strategies for how to prevent the formation of these pathological features. Obvious shortcomings of novel preventive drug development are based on the lack of knowledge of molecular upstream mechanisms, such as DNA methylation (DNAm) or gene expression (GE) (Perucca et al., 2020), and also the neglect of the importance of glial cell involvement in the inflammatory processes accompanying epileptogenesis (Kalozoumi et al., 2018; Patel et al., 2019). The goal of this review is to discuss the significance of DNAm as a potential target for anti-epileptogenic treatment in the scope of novel knowledge of glial involvement in this process.

## DNAm: Function and potential therapeutical target

After the human genome was deciphered in 2003, there were high expectations that pathological phenotypes would be linked to genomic variants (Collins et al., 2003; Green et al., 2015). However, the expectations did not come to fruition, and in the field of epilepsy, only a few genomic loci could be linked to epileptic conditions (International League Against Epilepsy Consortium on Complex Epilepsies, 2014; Abou-Khalil et al., 2018). The field of “epigenomics” arose with the hope to decipher the missing link between genotype and phenotype. Today, epigenetics can be defined as mechanisms that largely determine the “transcriptome,” meaning which genes are “used” to define a certain phenotype (Feinberg, 2007; McClung and Nestler, 2008; Gräff et al., 2011).

At the molecular level, epigenetics includes several mechanisms: DNAm, histone modifications, and non-coding RNAs are the most integral parts of this machinery (Gräff et al., 2011; Cavalli and Heard, 2019). These mechanisms are heavily interacted (Li et al., 2008; Cedar and Bergman, 2009; Wang et al., 2015). They determine chromatin accessibility, quality, and quantity of GE, and by this, tissue development in normal as well as pathological states. They can be altered by environmental factors (Danchin et al., 2011) and are mitotically inheritable (Cavalli and Heard, 2019). As such, they are potential upstream mechanisms for various molecular pathways, including those prompting pathological features of epileptogenesis. In the following, we will focus on DNA methylation in epileptogenesis.

DNA methylation is so far the most comprehensively studied epigenetic mechanism (Dor and Cedar, 2018) and is defined as the methylation (adding of a -CH3 group) of the DNA-base cytosine in a CpG (Cytosine – phosphate – Guanine) dinucleotide (CpG) (Luo et al., 2018). Oxidated variants of 5-methylcytosine (5-mC—in this review simply referred to as DNAm) include 5-hydroxymethylcytosine [5-hmC, relevant in post-mitotic neurons (Mellén et al., 2017)], 5-formylcytosine (5-fC), and 5-carboxylcytosine (5-caC), all of which potentially exert distinct effects on the GE and chromatin accessibility (Song and He, 2013). Other types of methylation include: non-CpG DNAm in, e.g., neurons (Kozlenkov et al., 2014), RNA methylation (Widagdo and Anggono, 2018), and mitochondrial DNAm (Cavalcante et al., 2020). In the central nervous system (CNS), DNAm has a major role in brain development, cell differentiation, and disease (Lister et al., 2013; Smith and Meissner, 2013; Sanosaka et al., 2017; Greenberg and Bourc'his, 2019). DNAm has been shown to influence the GE in a tissue-, context-, and cell-dependent manner (Smith and Meissner, 2013; Greenberg and Bourc'his, 2019), usually in a close interaction with transcription factors (Kribelbauer et al., 2019). How far DNAm states correlate with GE is still a matter of debate (Luo et al., 2018). GE is, on the other hand, regarded as an indicator of protein abundance and biological function, and provides a relevant estimate of downstream effects (Wang et al., 2009; Liu Y. et al., 2016; Silva and Vogel, 2016). Thus, DNAm might influence downstream molecular and cellular processes *via* GE regulation. DNAm is modifiable by environmental factors (Martin and Fry, 2018; Cavalli and Heard, 2019), hormones (Kovács et al., 2020), and even by neuronal activity (Guo et al., 2011). Recently, the site-specific modification of DNAm was shown to specifically alter GE (Liu X. S. et al., 2016; Liu and Jaenisch, 2019), which is especially interesting when it comes to novel therapeutic concepts. Further, disease-specific blood-DNAm pattern alterations have been reported in several pathological conditions (Fransquet et al., 2018; Agha et al., 2019; Henderson-Smith et al., 2019; Somineni et al., 2019) and proposed as possible biomarkers, with adjunct therapeutical implications (Kim et al., 2018; Berdasco and Esteller, 2019).

## DNAm in epilepsy

Epilepsy-related alterations in DNAm have been shown in previous studies in both animal models (Miller-Delaney et al., 2012; Kobow et al., 2013; Machnes et al., 2013; Ryley Parrish et al., 2013; Williams-Karnesky et al., 2013; Li et al., 2015; Lusardi et al., 2015; Debski et al., 2016; Liu X. et al., 2016; Zybura-Broda et al., 2016) and in humans (Zhu et al., 2012; Miller-Delaney et al., 2015; Liu X. et al., 2016; Zhang et al., 2021) (Table 1). The most consistent finding is a state of DNA hypermethylation occurring in chronic-epilepsy states in both animal models (Kobow et al., 2013) and human hippocampal tissues (Miller-Delaney et al., 2015). Although some studies associate alterations in DNAm with GE changes (Kobow et al., 2009; Li et al., 2015; Debski et al., 2016), a recent study was more hesitant to reach this conclusion (Lipponen et al., 2018).

**Table 1:** Relevant studies assessing DNA methylation (DNAm) in epileptogenesis.

**Species**	**Tissue/model**	**Methods**	**Results**	**Interpretation/conclusion**	**Reference chronol**.
Mouse	Contralateral HC/ intracortical KA	Glial and neuronal nuclei sorted by flow cytometry. Alterations in GE and DNAm were assessed with RRBS and RNAseq. R package edgeR was used for statistical analysis	The CLH features substantial, mostly cell-specific changes in both GE and DNAm in glia and neurons. Changes in GE overlapped to a great degree between CLH and ILH; changes in DNAm did not. A significantly lower number of glial genes up- and downregulated compared to previous results from the ILH (Berger et al., 2019). Several genes and pathways potentially involved in anti-epileptogenic effects were upregulated in the CLH.	The CLH displays substantial changes in GE and DNAm. GE changes related to potential anti-epileptogenic effects seem to dominate compared to the pro-epileptogenic effects in the CLH.	Berger et al., 2020
Human	Hippocampal tissue resected from patients with TLE-HS	Genome-wide CpG-DNAm profiling and RNAseq to Dprofile global changes in promoter methylation and GE in HS patients. Real time PCR was performed to validate the findings of DNAm and RNAseq.	A total of 16040 sites showed altered DNAm in all the CpG islands. Of these, 3185 sites were in the promoter regions, of which 66 genes showed an inverse correlation between DNAm and expression. These genes are largely related to pathways predicted to participate in axon guidance by semaphorins, MAPK, ionotropic glutamate receptor pathway, notch signaling, regulatory activities related to TFAP2A and immune response, with the most distinct ones included TFAP2A, NRP1, SEMA3B, CACNG2, MAP3K11, and ADAM17.	Collectively, findings implicate DNAm as a critical regulator of the pathogenic mechanisms of epileptogenesis associated with HS.	Dixi et al., 2020
Mouse	Ipsilateral HC/intracortical KA	Separation into neurons and glial nuclei was performed by flow cytometry. Changes in DNAm and GE were measured with RRBS and mRNAseq. R package edgeR for analysis.	Fulminant DNAm- and GE changes in both neurons and glia at 24 hours after initiation of status epilepticus were observed. The vast majority of these changes were specific for either neurons or glia. At several epilepsy-related genes, like *Hdac11, Spp1, Gal, Drd1 and Sv2c*, significant differential DNAm and differential GE coincided.	Neuron- and glia-specific changes in DNAm and GE in early epileptogenesis. Single genetic loci in several epilepsy-related genes, where DNAm and GE changes coincide, were detected.	Berger et al., 2019
Human	Focal cortical dysplasia (FCD)	DNA methylomes and transcriptomes were generated from massive parallel sequencing in 15 surgical FCD specimens, matched with 5 epilepsy and 6 non-epilepsy controls.	Differential hierarchical cluster analysis of DNAm distinguished major FCD subtypes (ie, Ia, IIa, and IIb) from patients with temporal lobe epilepsy patients and nonepileptic controls. Targeted panel sequencing identified a novel likely pathogenic variant in DEPDC5 in a patient with FCD type IIa. However, no enrichment of differential DNAm or GE was observed in mechanistic target of rapamycin (mTOR) pathway-related genes.	Evidence for disease-specific DNAm signatures toward focal epilepsies in favor of an integrated clinicopathologic and molecular classification system of FCD subtypes incorporating genomic DNAm.	Kobow et al., 2019
Human	Focal cortical dysplasia (FCD)	Genome-wide CpG-DNAm profiling by methylated DNA immunoprecipitation (MeDIP) microarray and RNAseq on cortical tissues resected from FCD type II patients.	A total of 19088 sites showed altered DNAm in all the CpG islands. Of these, 5725 sites were present in the promoter regions, of which 176 genes showed an inverse correlation between DNAm and GE. Many of these 176 genes were found to belong to a cohesive network of physically interacting proteins linked to several cellular functions. Pathway analysis revealed significant enrichment of receptor tyrosine kinases (RTK), EGFR, PDGFRA, NTRK3, and mTOR signaling pathways	The first study investigating the epigenetic signature associated with FCD type II pathology. Identified candidate genes may play a crucial role in the regulation of the pathogenic mechanisms of epileptogenesis associated with FCD type II pathologies.	Dixi et al., 2018
Human	Blood	Comparison of blood whole genomic DNAm pattern in MTLE patients (n = 30) relative to controls (n = 30) with the Human DNAm 450 K BeadChip assay, exploring genes and pathways that are differentially methylated using bioinformatics profiling.	MTLE and control groups showed significantly different DNAm at 216 sites, with 164 sites involved hyper- and 52 sites hypo- DNAm. Two hyper- and 32 hypo-methylated sites were associated with promoters, while 87 hyper- and 43 hypo-methylated sites corresponded to coding regions. Differentially methylated genes were largely related to pathways predicted to participate in anion binding, oxidoreductant activity, growth regulation, skeletal development and drug metabolism, with the most distinct ones included *SLC34A2, CLCN6, CLCA4, CYP3A43, CYP3A4* and *CYP2C9*. Panels of genes also appeared to be differentially methylated relative to disease duration, resistance to anti-epileptics and MRI alterations of HS.	The peripheral epigenetic changes observed in MTLE could be involved in certain disease-related modulations and warrant further translational investigations.	Long et al., 2017
Human	Brain tissue from refractory epilepsy patients	Genome-wide DNAm and GE in brain tissues of 10 patients with refractory epilepsy using methylated DNA immunoprecipitation linked with sequencing and mRNAseq.	Diverse distribution of differentially methylated genes was found in X chromosome, while differentially methylated genes appeared rarely in Y chromosome. 62 differentially expressed genes, such as *MMP19, AZGP1, DES*, and *LGR6* were correlated with refractory epilepsy.	Findings provide a genome-wide profiling of DNAm and GE in brain tissues of patients with refractory epilepsy, which may provide a basis for further study on the etiology and mechanisms of refractory epilepsy.	Liu X. et al., 2016
Rat	3 models: - focal amygdala stimulation/ - systemic pilocarpine/ - lateral fluid-percussion (TBI)	DNAm and GE in the hippocampal CA3/dentate gyrus fields at 3 months following epileptogenic injury in three experimental models.	DNAm and GE profiles distinguished ctr. from injured animals. Consistent increased DNAm in gene bodies and hypoDNAm at non-genic regions. A common DNAm signature for all three different models was not found, and few regions common to any two models.	Evidence that genome-wide alteration of DNAm signatures is a general pathomechanism associated with epileptogenesis and epilepsy in animal models, but the broad pathophysiological differences between models are reflected in distinct etiology-dependent DNAm patterns.	Debski et al., 2016
Human	Brain tissue from epilepsy patients and ctr.	DNAm via methylated-cytosine DNA immunoprecipitation microarray chip. Differentially methylated loci validated by bisulfite sequencing PCR, and mRNA levels of candidate genes evaluated by RT-PCR.	224 genes showed differential DNAm between epileptic patients and ctr. Among the seven candidate genes, three genes (*TUBB2B, ATPGD1*, and *HTR6)* showed relative transcriptional regulation by DNAm. *TUBB2B* and *ATPGD1* exhibited hyper DNAm and decreased mRNA levels, whereas *HTR6* displayed hypo DNAm and increased mRNA levels in the epileptic samples.	Findings suggest that certain genes become differentially regulated by DNAm in human epilepsy.	Wang et al., 2016
Human	Resected HC tissue from TLE with- or without HS	DNAm analysis of all annotated CpG islands and promoter regions in the human genome. Comparative analysis of expression and promoter DNAm	146 protein-coding genes exhibited altered DNAm in TLE hippocampus (n = 9) when compared to ctr. (n = 5), with 81.5% of the promoters displaying hyper DNAm. Unique DNAm profiles were evident in TLE with or without HS, in addition to a common DNAm profile regardless of pathology grade.	The present study therefore reports select, genome-wide DNAm changes in human temporal lobe epilepsy that may contribute to the molecular architecture of the epileptic brain.	Miller-Delaney et al., 2015
			GO terms associated with development, neuron remodeling and neuron maturation were over-represented in the DNAm profile of mild HS. In addition to genes associated with neuronal/synaptic transmission and cell death functions, differential hyperDNAm of genes associated with transcriptional regulation in TLE. A panel of 13, DNAm-sensitive microRNA are identified in TLE including *MIR27A*, miR-193a-5p (*MIR193A*) and miR-876-3p (*MIR876*), and the differential DNAm of long non-coding RNA.		
Mouse/Rat	Transient kainic acid exposure using *in vitro* and *in vivo* rodent models.	Analysis of DNAm changes in the gria2 gene, which encodes for the GluA2 subunit of the ionotropic glutamate, alpha-amino-3-hydroxy-5-methyl-4-isoxazole proprionic acid receptor	KA exposure for 2 h to mouse hippocampal slices triggers DNAm of a 5' regulatory region of the gria2 gene. Increase in DNAm persists one week after removal of the drug, with concurrent suppression of gria2 mRNA expression levels. In a rat *in vivo* model of post kainic acid-induced epilepsy, we show similar hyperDNAm of the 5' region of gria2. Luciferase reporter assays support a regulatory role for DNAm of gria2 5' region. Inhibition of DNAm by RG108 blocked KA-induced hyperDNAm of gria2 5' region in HC slice cultures.	Our results suggest that DNAm of such genes as gria2 mediates persistent epileptiform activity and inter-individual differences in the epileptic response to neuronal insult and that pharmacological agents that block DNAm inhibit epileptiform activity raising the prospect of DNAm inhibitors in epilepsy therapeutics.	Machnes et al., 2013
Rat	Rat brain specimens	Methyl-CpG capture associated with massive parallel sequencing (Methyl-Seq) to assess the genomic DNAm. mRNAseq for GE analysis	Predominant increase of DNAm in chronic rat epilepsy. Aberrant DNAm patterns were inversely correlated with GE changes using mRNAseq from same animals and tissue specimens. Administration of a ketogenic, high-fat, low-carbohydrate diet attenuated seizure progression and ameliorated DNAm mediated changes in GE.	First report of unsupervised clustering of an epigenetic marker being used in epilepsy research to separate epileptic from non-epileptic animals as well as from animals receiving anti-convulsive dietary treatment.	Kobow et al., 2013
Rat	Experimental TLE provoked by kainic acid-induced SE	Bisulfite sequencing analysis; chromatin immunoprecipitation analysis	Increased glutamate receptor subunit epsilon-2 (*Grin2b/Nr2b*) and decreased *Bdnf* DNAm levels that corresponded to decreased *Grin2b/Nr2b* and increased *Bdnf* mRNA and protein expression in the epileptic hippocampus. Blockade of DNA methyltransferase (DNMT) activity decrease global DNAm levels and reduced *Grin2b/Nr2b*, but not *Bdnf* DNAm levels; and decreased *Grin2b/Nr2b* mRNA expression whereas GRIN2B protein expression increased in the epileptic hippocampus, suggesting that a posttranscriptional mechanism may be involved.	DNAm may be an early event triggered by SE that persists late into the epileptic hippocampus to contribute to GE changes in TLE.	Ryley Parrish et al., 2013
Mouse	Hippocampus	Genome-wide DNAm analysis of 34,143 discrete loci representing all annotated CpG islands and promoter regions in the mouse genome	321 genes showed altered DNAm after status epilepticus alone or status epilepticus that followed seizure preconditioning, with >90% of the promoters of these genes undergoing hypo DNAm. These profiles included genes not previously associated with epilepsy, such as the polycomb gene *Phc2*. Differential DNAm events occurred throughout the genome, with the exception of a small region of chromosome 4, which was significantly overrepresented with genes hypomethylated. Gene ontology analysis emphasized the majority of differential DNAm events between the groups occurred in genes associated with nuclear functions, such as DNA binding and transcriptional regulation.	Evidence for genome-wide DNAm changes after status epilepticus and in epileptic tolerance, which may contribute to regulating the GE environment of the seizure-damaged hippocampus.	Miller-Delaney et al., 2012
Human	Hippocampus	DNA from 3 dissected HC regions from MTS specimens with granular cell dispersion (GCD), TLE samples without GCD, and autopsy ctrs. Promoter DNAm analyzed after bisulfite treatment, cloning, and direct sequencing	*Reelin* promoter DNAm was found to be greater in TLE specimens than in ctrs; promoter DNAm correlated with GCD among TLE. No other clinical or histopathological parameter (i.e. sex, age, seizure duration, medication or extent, of MTS) correlated with promoter DNAm.	These data support a compromised Reelin-signaling pathway and identify promoter DNAm as an epigenetic mechanism in the pathogenesis of TLE.	Kobow et al., 2009

At the early stages of epileptogenesis, no changes (Ryley Parrish et al., 2013), or a slight tendency toward general DNA hypomethylation (Miller-Delaney et al., 2012), have been found. At single genes, DNAm changes have been recorded as soon as 1 h after status epilepticus (SE) initiation by intra-peritoneal (i.p.) kainate treatment in rats (Ryley Parrish et al., 2013). At chronic time points, a general tendency toward hypermethylation was found, and potential associations between DNAm and GE at specific genomic loci identified (Kobow et al., 2013). A follow-up study reanalyzed these results and compared them to two other murine models of focal epilepsy, traumatic brain injury (TBI) and amygdala stimulation, at chronic time points. This study found both a general tendency toward CpG hypomethylation (amygdala stimulation) and hypermethylation (pilocarpine, TBI) methylation and little overlap regarding DNAm between these models (Debski et al., 2016).

Some studies investigated both DNAm and GE changes to identify potential correlations. Although no general correlations between DNAm and GE were found, coincidental occurrences of these two phenomena were detected at some genomic loci (Kobow et al., 2013; Debski et al., 2016). Other studies have used more of a “black box” approach, investigating general methylation levels and testing interventions by means of DNA-methyl-transferase inhibitors—targeting the “hypermethylated” state in chronic epilepsy and potentially attenuating epileptogenesis. In one study (rat model, i.p. kainate) the application of a DNMT inhibitor did not significantly alter the disease course (some/despite of later onset of SE), but it did reduce the general hypermethylation state and also DNAm at one of the investigated genes (Ryley Parrish et al., 2013). Another group reported reversed hypermethylation, attenuated seizure severity, and later onset of epileptogenesis (kindling model in mice and rats using pentylenetetrazol) associated with both the application of a DNMT inhibitor and adenosine (intracranial implants) (Williams-Karnesky et al., 2013). In our previous studies employing the intracortical kainate mouse model of mesial temporal lobe epilepsy, we observed vast and mainly cell-specific changes in DNAm in both the ipsilateral (Berger et al., 2019) and contralateral (Berger et al., 2020) hippocampi, with hypermethylation generally outweighing hypomethylation in both hippocampi. DNAm alterations near epilepsy-relevant genes and genes within epilepsy relevant Gene Ontology/Kyoto Encyclopedia of Genes and Genomes (GO/KEGG) pathways (Berger et al., 2019, 2020). However, we did not detect general correlations between DNAm and GE on genomic features (Berger et al., 2019, 2020). Nevertheless, at several potentially epileptogenesis-related genes, statistically significant alterations in DNAm and GE coincided (Berger et al., 2019). In summary, in this specific model, we were not able to conclude that DNAm facilitates a direct regulation of GE in early epileptogenesis. This conclusion concurs with results from work on the potential role of DNAm for GE in epileptogenesis (Lipponen et al., 2018) and the theory that DNAm mostly represents a secondary molecular marker of long-term gene silencing (Dor and Cedar, 2018; Luo et al., 2018; Greenberg and Bourc'his, 2019). In addition, prior studies investigating general methylation trends or associations have not demonstrated mechanistic correlations between DNAm and GE/protein alterations/epileptogenesis, but rather have shown co-incidences. Nevertheless, many previous studies and our data suggest wide-spread changes in DNAm in epileptogenesis. As the different epigenetic mechanisms (histone modifications and microRNA [miRNA]) are inter-linked (Li et al., 2008; Cedar and Bergman, 2009; Wang et al., 2015), DNAm may still affect downstream effects, not necessarily directly, but indirectly.

## Increasing detection sensitivity by cell-specific approaches including focus on glial cells

Several recent studies have revealed a possible involvement of DNAm in both epileptogenesis and GE (Table 1). However, reports are not consistent and possible shortcomings may be associated with the widely used simultaneous bulk analysis of several different CNS cell types. The analysis of DNAm and GE in neurons and glia cells individually may rectify a crucial shortcoming of previous studies and be one prudent step toward deciphering the “DNA methylome.” Neurons and glia facilitate mostly complementary tasks in physiological and pathological CNS states, such as epileptogenesis (Cahoy et al., 2008; Doyle et al., 2008; Zamanian et al., 2012; Patel et al., 2019), and exhibit different DNAm methylation profiles (Kozlenkov et al., 2014; Sanosaka et al., 2017). DNAm both in ways of 5hmC and non-CpG methylation are of a much higher significance in neurons than in glial cells (Kozlenkov et al., 2014; Mellén et al., 2017). Analyzing molecular mechanisms in these individual cell types separately enhances resolution, and information can be obtained about the cellular origin of detected effects (e.g., changes in DNAm and GE, as well as their possible correlation). Further, the methylation of several genomic regions, such as regulatory elements exerts cell-specific effects (Li et al., 2021).

We recently introduced a more cell-specific methodology in 2 separate publications using cell sorting of brain tissue into neuronal and non-neuronal (glial) cells prior to DNAm and GE analysis (Berger et al., 2019, 2020). We found considerable changes in both DNAm and GE at 24 h post-initiation of SE in a mouse model of mesial temporal lobe epilepsy with hippocampal sclerosis (Berger et al., 2019, 2020). Most of these molecular alterations were specific to either neurons or glia. Further, GE changes uncovered a substantial involvement of glia cells in processes crucial to epileptogenesis, such as inflammation, neuronal death, neurogenesis, and Ca^2+^ signaling. Furthermore, we found a substantial overlap of genomic dysregulation with other epilepsy models, and even with other neurodegenerative diseases, such as Parkinson's disease or multiple sclerosis (Berger et al., 2019, 2020). These studies underscored our postulation that a cell-specific analysis of DNAm and GE in epileptogenesis provides deeper knowledge about the cellular origin of molecular mechanisms and by this, it provides a clearer view on putative upstream targets for drug development. This makes expressly sense in the scope of novel knowledge on glial cells as important orchestrators of brain inflammation and epileptogenesis. In the following, we focus on differentially regulated glial genes and their probable contribution to epileptogenesis.

## Glial contribution to epileptogenesis

With our novel understanding of glial cells as central organizers of homeostatic functions and as major contributors to inflammation and brain excitability, we observe a paradigm shift where glial cells are included in the equation of epilepsy pathogenesis. Through this, we expect to approach novel curative treatment strategies for epilepsy (Heuser et al., 2021).

In the following, the most prominent hallmarks of epileptogenesis and specifically their glial contribution are discussed in detail, as alteration of glia-mediated downstream effects may represent novel treatment targets for anti-epileptogenic intervention.

### Differentially regulated glial genes and their involvement in neuronal death

Death of pyramidal neurons in CA3/1 is a major hallmark of human mTLE-HS (Blumcke et al., 2013) and is reproduced in many animal models, including the intracortical kainic acid mouse model of mTLE-HS (Bedner et al., 2015). In this now widely used and well-characterized model, apoptosis is first detectable after 6 h post-injection (hpi)—but not at 4 h—in CA1 neurons (Bedner et al., 2015). At 24 hpi, both CA1 and CA3 exhibit apoptotic pyramidal neurons, and, from then on, a progressive neurodegeneration leads eventually to 90% neuronal death in CA1 and CA3 at 28 days post-injection (dpi), and a complete absence of hippocampal neurons at 9 months post-injection (mpi) (Bedner et al., 2015). Further, the majority of GABAergic interneurons in CA1 and the dentate gyrus responsible for tonic inhibition are diminished substantially already at 5 dpi (Müller et al., 2020). At 24 hpi, we detected that 8 genes related to the regulation of neuronal death were upregulated in glia, while 2 genes in this category were downregulated in neurons (Berger et al., 2019). Many of these glial genes are associated with tumor necrosis factor alpha (TNF-α), nuclear factor kappa-light-chain-enhancer of activated B cells (NF-kB), and interleukin 1 beta (IL-1β) related pathways (Berger et al., 2019) and have previously been shown to be upregulated to some extent in microglia, but mostly astrocytes, in reactive states (Schlomann et al., 2000; Almeida et al., 2005; Koyama and Ikegaya, 2005; Saha et al., 2006; Groves et al., 2018; Iughetti et al., 2018). Although these results do not necessarily imply a causal role, they at least indicate an important involvement of glia in general, and astrocytes in particular, for neuronal death and already in the early latent phase of epileptogenesis.

### Differentially regulated glial genes and their involvement in reactive astrogliosis

Reactive astrogliosis is a common feature of various neurodegenerative states (Zamanian et al., 2012) and pathognomonic for several epileptic conditions, such as mesial temporal lobe epilepsy with hippocampal sclerosis (mTLE-HS) in humans, successfully mimicked in the i.c. mouse model of mTLE-HS (Blumcke et al., 2013; Bedner et al., 2015). In general, this phenomenon comprises morphological and molecular reshaping of astrocytes in response to an external stimulus, and it is associated with astrocyte proliferation, immune-cell recruitment, and scar formation (Escartin et al., 2021). These responses are not necessarily dichotomous as previously proposed (good vs. bad astrocytes) (Zamanian et al., 2012), but represent a continuum of various molecular responses, the cumulative consequences of which are at best unknown (Escartin et al., 2021).

In mTLE-HS, astrocytes undergo functional and morphological changes already in the early stages of epileptogenesis. As shown in the i.c. mouse model, already at 4 hpi, cell death (primarily *via* necroptosis and autophagy) is detectable in astrocytes and the number of astrocytes in the CA1 region is reduced (Wu et al., 2021). These structural alterations are accompanied by decreased coupling and impaired capability of K+ buffering (Bedner et al., 2015; Wu et al., 2021), which in turn are associated with increased extracellular glutamate levels and epileptic seizures (Pannasch et al., 2011). Over the course of epileptogenesis, astrocytes proliferate, express more GFAP, and at 9 mpi, a time point relevant to findings in humans with mTLE-HS, clear structural integration into the surrounding tissue is absent (Bedner et al., 2015). Our data at 24 hpi in the ipsilateral hippocampus (Berger et al., 2019), reveal that *Serpina3n* is the most significantly differentially expressed gene in glia. This astrocytic gene is associated with inflammation (Takamiya et al., 2002) and neuronal damage (Gesase and Kiyama, 2007) and has previously been identified as one of the major molecular hallmarks of reactive astrogliosis (Zamanian et al., 2012). Other interesting genes upregulated at 24 hpi in the ipsilateral hippocampus are *Cox2* (*Ptgs2*) and *Cxcl10* (Berger et al., 2020), both of which are pro-epileptic and potentially pro-epileptogenic inflammatory agents (Nelson and Gruol, 2004; Sui et al., 2006; Rojas et al., 2014) that are dysregulated in reactive astrogliosis (Zamanian et al., 2012). Other potential pathways involved in functional astrocytic alterations, such as astrocyte uncoupling, are the upregulation of mitogen-activated protein kinase (MAPK) pathways observed at 24 hpi mainly in glia (Berger et al., 2019). Early astrocytic uncoupling has been linked to altered phosphorylation of Cx43 *via* MAPK (Deshpande et al., 2017). This potentially involves TNF-α and IL-1β (Retamal et al., 2007) [mostly increased in glia (Berger et al., 2019)], which have been previously shown to have a negative effect on astrocyte coupling in the i.c. mouse model of mTLE-HS (Bedner et al., 2015).

### Differentially regulated glial genes and their involvement in brain inflammation

Inflammation is closely associated with epileptogenesis (Vezzani et al., 2011). Both astrocytes and microglia can initiate and modulate inflammatory responses (Aronica et al., 2012; Devinsky et al., 2013; Eyo et al., 2017; Liddelow et al., 2017) and orchestrate downstream alterations, such as neuronal death or reactive gliosis (Jha et al., 2019; Patel et al., 2019). Apart from the activation of astrocytes at early time points of epileptogenesis in the i.c. mouse model of mTLE-HS, microglia are both prone to necroptosis already at 4 hpi and activated (elevated Iba1) at 14 dpi (Deshpande et al., 2020; Wu et al., 2021). Elevations in the levels of both TNF-α and IL-1β have also been measured at the early stages of epileptogenesis in this model. At 24 hpi, we observed increased TNF-α and IL-1β pathways, mainly in glia (Berger et al., 2019). Both these cytokines effect glutamate uptake and release from astrocytes (Hu et al., 2000; Bezzi et al., 2001; Santello et al., 2011). They also affect astrocyte coupling and neuronal death. Inflammation is also connected to the disruption of the BBB, angiogenesis, alterations of the ECM, and aberrant neurogenesis, all important elements of epileptogenesis (Pitkanen and Lukasiuk, 2011; Patel et al., 2019). In the i.c. kainic acid model of mTLE-HS, albumin extravasation is detected at 5 dpi and throughout epileptogenesis (3 and 9 mpi) (Deshpande et al., 2017). At 28 dpi, CD31 as a marker of endothelial cells, and as such of angiogenesis, is increased in the ipsilateral hippocampus 3-fold in both CA1 and DG (Deshpande et al., 2017). This is in line with our findings in the same model, where we at 24 hpi, detected a total of 19 glial genes and 11 neuronal genes involved in angiogenesis (Berger et al., 2019). Albumin extravasation is known to induce the activation of transforming growth factor beta (TGF-β) in astrocytes (Heinemann et al., 2012), which, in turn induces MAPKs potentially leading to Cx43 phosphorylation and uncoupling of astrocytes, a mechanism believed to be important in epileptogenesis (Deshpande et al., 2017). TGF-β further induces increased neuronal excitability *via* astrocyte-mediated reduction of Kir4.1, AQP channels, and glutamate transporters (Ivens et al., 2007; Kim et al., 2012). We found TGF-β pathways to be increased in both neurons and glia at 24 hpi (Berger et al., 2019).

## Potential strategies and challenges of anti-epileptogenic intervention

Hypermethylation in chronic epilepsy states in both human and murine CNS tissue, represents the most consistent finding of DNAm alterations. Alas, the potential attenuation of epileptogenesis *via* DNAm inhibition could serve as an anti-epileptic or even anti-epileptogenic target (Ryley Parrish et al., 2013; Williams-Karnesky et al., 2013). Mechanistically, this may be linked to direct anti-epileptic/anti-epileptogenic effects of adenosine, as this molecule is metabolically connected to DNAm (Weltha et al., 2019).

Anti-epileptogenic therapy could either enforce endogenic homeostatic pathways or attenuate detrimental responses. Genes dysregulated in the latent phase but also in the early chronic state of epileptogenesis are potential upstream targets of anti-epileptogenic intervention. In general, all the above-mentioned genes with CpG islands in their promoters (possibly other genomic features also) could be targeted to alter GE *via* DNAm alteration [as shown feasible in ref. (Liu X. S. et al., 2016)]. Even if DNAm changes do not overlap with GE changes in different epilepsy models and at different specific time points, it may still be possible to alter the expression of crucial genes and pathways by means of epigenetic editing as described elsewhere (Liu X. S. et al., 2016; Holtzman and Gersbach, 2018; Liu and Jaenisch, 2019). To achieve this goal, an epigenetic tool, such as a modified CRISPR system with either a DNMT (to facilitate hypermethylation and potentially gene silencing) or a TET oxidase (to gain hypomethylation and gene activation), applicable both to the anatomical region and cell type of choice, would be necessary.

Further, specific post-transcriptional (Desi and Tay, 2019) or post-translational modifications (Wang et al., 2014) could be employed to intervene between gene transcription and protein synthesis/downstream effects of the given target. As proposed by previous studies and supported by our findings (Berger et al., 2019, 2020), glia plays an important role in inflammatory pathways (Devinsky et al., 2013; Patel et al., 2019). Thus, the development of glia-specific drugs may lead the way to the next generation of anti-epileptogenic treatment. Nevertheless, a long way is ahead to reach these goals. One way to decipher the “epileptogenic code” would be to analyze all known molecular factors (e.g., DNAm, histone modifications, transcription factor binding, GE, proteomics, EEG, and clinical parameters) simultaneously and using comparable methods, crystalize patterns with the help of artificial intelligence, as has been performed with a fraction of parameters in ref. (Myszczynska et al., 2020). The next pragmatic steps toward this long-term goal include the assessment of cell-specific DNAm and throughout the whole process of epileptogenesis in animal models, with confirmation of results in human studies. Potential outcomes of such efforts include cell-specific molecular treatment targets and biomarkers for epileptogenesis. However, one of the most prominent challenges is the versatility of the epilepsies. Seizures are symptomatic manifestations of cerebral dysfunction and can be caused by a multitude of conditions, ranging from unknown idiopathic to the acquired causes, such as TBI, ischemic cerebral insults, infections, or hypoxia. Moreover, we must rely on simplified models restricted to only mimicking a certain group of the human epilepsies. Further, we must face concerns on comparability, interpretability, and translation of various research results based on diverse analytical tools (e.g., for GE—RNA-Seq or various kits) and bioinformatics processing.

Another important issue worth mentioning is the role of DNAm for diagnostic and prognostic approaches in epilepsy. As an example, the blood of patients with mTLE-HS has shown specific DNAm patterns (Long et al., 2017) and a concordant-twin study detected patterns that enabled distinction between focal and generalized epilepsy (Mohandas et al., 2019). These results suggest that blood-based DNAm could be a potential biomarker for epilepsy and epileptogenesis, paving the way to a more personalized management of people with epilepsy (PWE). Blood-based DNAm as a biomarker could also help to predict the patients risk for developing comorbidities, such as anxiety, depression, or cognitive decline and predict treatment response with existing anti-seizure medication (ASM).

## Conclusions

Neurons and glia orchestrate epileptogenesis, with glia playing a crucial part in neuronal death, reactive gliosis, and inflammation in early epileptogenesis. Several, mostly inflammation-related and glia-derived genes may serve as targets for anti-epileptogenic intervention, potentially by means of epigenetic modification. Several recent studies have revealed widespread and cell-specific alterations in DNAm in epileptogenesis. To what extent and how exactly DNAm influences GE (and downstream effects) in epileptogenesis, is challenging to determine, and methodological shortcomings as well as inadequate processing of complex data seem to muddy the waters. We have yet to fathom the full complexity of interplay at various levels of molecular interactions in epileptogenesis. It is possible, or even likely, that not a single gene or pathway determines epileptogenesis, but rather the interaction of genes at a given time, the resulting levels of proteins, and cellular interactions that determine the epileptogenic phenotype.

## Author contributions

KH and TB contributed to conception, design, writing, and revision of the article. ET contributed to the writing and revision of the article. All authors contributed to the article and approved the submitted version.

## Funding

This work was supported by the European Commission (ERA-NET NEURON, Brain Inflammation, Glia and Epilepsy), the European Union's Horizon 2020 research and innovation program (Marie Sklodowska-Curie grant agreement No. 722053), and the South-Eastern Norway Regional Health Authority (No. 2014018).

## Conflict of interest

The authors declare that the research was conducted in the absence of any commercial or financial relationships that could be construed as a potential conflict of interest.

## Publisher's note

All claims expressed in this article are solely those of the authors and do not necessarily represent those of their affiliated organizations, or those of the publisher, the editors and the reviewers. Any product that may be evaluated in this article, or claim that may be made by its manufacturer, is not guaranteed or endorsed by the publisher.
